# Absorption coefficient of carbon dioxide across atmospheric troposphere layer

**DOI:** 10.1016/j.heliyon.2018.e00785

**Published:** 2018-10-06

**Authors:** Peng-Sheng Wei, Yin-Chih Hsieh, Hsuan-Han Chiu, Da-Lun Yen, Chieh Lee, Yi-Cheng Tsai, Te-Chuan Ting

**Affiliations:** Department of Mechanical and Electro-Mechanical Engineering, National Sun Yat-Sen University, Kaohsiung 80424, Taiwan, ROC

**Keywords:** Atmospheric science, Environmental science, Geophysics

## Abstract

Absorption coefficient affected by carbon dioxide concentration and optical path length responsible for temperature or global warming across the troposphere layer, which is less than the altitude of 10 km in the atmosphere, is systematically presented in this work. Solar irradiation within a short wavelength range can be absorbed, scattered and transmitted by the atmosphere, and absorbed and reflected by the Earth's surface. Radiative emission in high wavelength ranges from the Earth's surface at low temperature can be absorbed by atmospheric water vapor, carbon dioxide and other gases. Unbalance of radiation thus results in the atmosphere to act as the glass of a greenhouse and increase atmospheric temperature. Even though global warming strongly affects the life of the human being, the cause of global warming is still controversial. This work thus proposes a fundamental and systematical unsteady one-dimensional heat conduction-radiation model together with exponential wide band model to predict absorption coefficients affected by concentration, temperature, optical path lengths and radiation correlated parameters in different bands centered at 15, 4.3, 2.7, and 2 μm of carbon dioxide across the troposphere layer. It shows that absorption coefficient required for calculating heat transfer is strongly affected by carbon dioxide concentration and optical path length across the troposphere. Relevant values of the latter should be greater than 5,000 m. Absorption coefficients in the band centered at 4.3 μm subject to a chosen optical path length of $10^{4}$ m increase from 0.04 m^−1^ and 0.165 m^−1^at the tropopause to 0.11 m^−1^ and 0.44 m^−1^ at the Earth's surface for carbon dioxide concentrations of 100 and 400 ppm, respectively. A more relevant and detailed temperature profile across the troposphere is presented.

## 1 Introduction

Radiation heat transfer due to carbon dioxide plays an important role in the greenhouse effect, climate change, and global warming. The Intergovernmental Panel on Climate Change (IPCC) from the United Nations concluded that the greenhouse gas emission (particularly carbon dioxide) due to the burning of fossil fuels carbon dioxide is responsible for climate change and global warming. The first estimates of how changes in the global concentration of carbon dioxide might affect mean global surface temperature were made by Arrhenius [1]. He demonstrated that an increase in the atmospheric concentration of carbon dioxide by a factor of two would lead to a heating of the earth's temperature by 5–6 °C. Callendar [2] demonstrated through laboratory experiments that increased carbon dioxide concentration could have significant global effects on the surface temperature of the earth. It was also speculated for the first time that humans could have a significant influence on the atmospheric carbon dioxide concentration. Complicated climate change issues thus have led to considerable controversy in scientific commentaries and articles [3, 4, 5, 6, 7, 8], books and monographs [9, 10, 11], describing the factors of human activities (IPCC, 1996), solar variability [12, 13], cloud cover [14, 15, 16, 17] and aerosols [18], biosphere [19], urbanization and land-use change [8], volcanism [20], magnetism [21], etc.

**Fig. 1:**
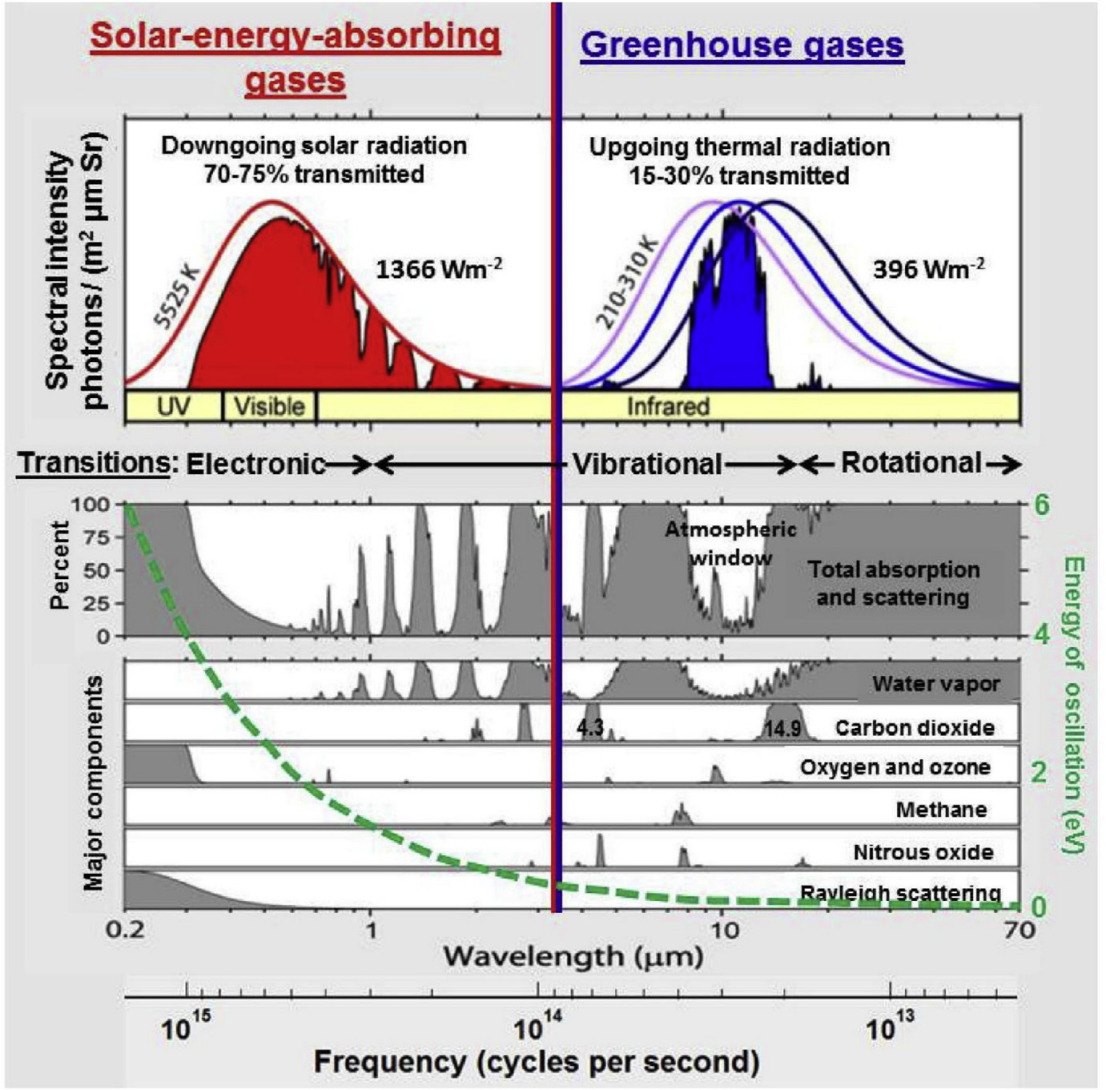
Transmission of shortwave solar irradiation and longwave radiation from the Earth's surface through atmosphere [22]. Greenhouse effect results from difference in downgoing solar radiation in red region and upgoing thermal radiation in blue region. Solar irradiation is absorbed, scattered and transmitted through the atmosphere and absorbed by the Earth's surface. Irradiation from the Earth's surface can be absorbed by carbon dioxide, water vapor and other emission gases in different bands of wavelength. Major amounts of oxygen gas and nitrogen gas are transparent to infrared radiation.

Absorption responsible for greenhouse effect depends on not only temperature and concentration of emission gases but also wavelength. As illustrated in Fig. 1 [22], greenhouse effect results from difference in downgoing solar radiation in red region and upgoing thermal radiation in blue region. Solar irradiation in visible range of short wavelength is absorbed, scattered and transmitted through the atmosphere and absorbed by the Earth's surface. In view of low temperature, the Earth's surface radiates energy with long wavelength. Radiation can be absorbed by carbon dioxide, water vapor and other emission gases in different bands or ranges of wavelength. Absorption bands of carbon dioxide are centered at 15, 4.3, 2.7, and 2 μm. There exists a window between 8 and 14 μm for absorption bands of water vapor [23]. Infrared absorption and emission of thermal radiation is a consequence of coupled vibrational and rotational energy transitions. Polyatomic molecules such as carbon dioxide and water vapor undergo such transitions. Major amounts of oxygen gas and nitrogen gas are transparent to infrared radiation. This is because they are symmetric diatomic molecules without permanent dipole moment.

To clarify uncertain processes involved in global warming, a fundamental, systematical and detailed study of absorption across the troposphere layer is essentially required. This leads to solving the radiative transfer equations together with relevant values and less computational demanding of radiative properties. One possibility of obtaining more simplicity for spectral modelling is the calculation of one value for every spectral band. These types of models are the wide band models. Excellent reviews can be seen in Modest [23], Howell et al. [24], Edwards [25], Cess and Tiwari [26], Tien [27], etc. The total band absorption A was first measured extensively for atmospheric carbon dioxide for different pressures and absorber concentration of carbon dioxide and water vapor at 300 K by Howard et al. [28, 29, 30]. Since a narrow band has an absorptivity linear in mass path length, X, at low values and square root at higher ones, the total band absorptance A would be linearly with X at low values, with $X^{1/2}$ at moderate values. A logarithmic relation was used for highest experimental values. There are thus theoretical bases for correlating and constructing more accurate and simple wide-band models. Marin and Buckius [31], Chu and Greif [32], Lin and Greif [33], Edwards and Balakrishnan [34], Hsieh and Greif [35], Tien and Lowder [36], Cess and Wang [37], et al., have explored such theoretically and successfully based models. Undoubtedly progress has become both possible and convenient to base engineering calculations. A further successful model for wide band absorption been shown by Edwards and Menard [38]. The Goody model for spectral absorptivity as a function of intensity-to-line-spacing and line-width-to-spacing ratios was used to construct band models. Total band absorption vs mass path length and pressure was then obtained for each so-constructed model by integration over wave number. Asymptotic relations for the total band absorption vs mass path length and pressure obtained from each model are successfully compared and found to be linear, square root, square root logarithmic, and logarithmic relations.

In this work, absorption coefficients of carbon dioxide used for a fundamental and quantitative study of heat transfer across the troposphere atmosphere are systematically determined. Absorption coefficients in different bands are calculated from the exponential wide band model [23, 24, 34] with temperature profile obtained by solving heat conduction and radiation equations. This study provides a first step, fundamental and systematic understanding of greenhouse effect and global warming phenomena.

## 2 Model

**Fig. 2:**
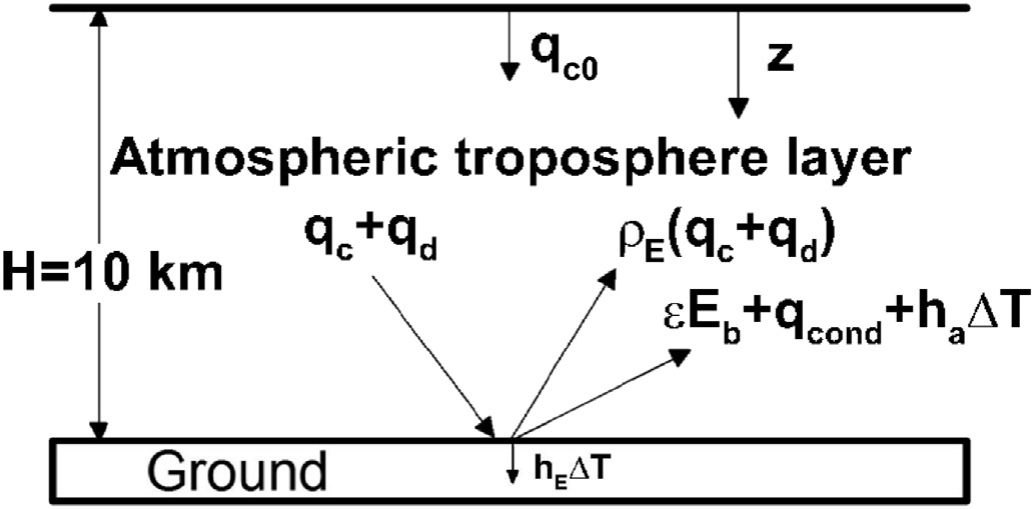
Schematic of the physical model. Solar irradiation imposed at the tropopause z = 0 is absorbed, scattered and transmitted through the troposphere and absorbed by the Earth's surface. The Earth's surface radiates energy with long wavelength. Collimated and diffuse irradiation and their reflections, heat conduction and convection occur on the Earth's surface at z = H.

In this work, the physical domain is the troposphere layer on the Earth's surface, as illustrated in Fig. 2. The z coordinate measured from the top (namely, tropopause) is directed toward the Earth's surface at z = H. Solar irradiation with small wavelengths imposed at the tropopause is absorbed, scattered and transmitted through the troposphere and absorbed by the Earth's surface. In view of low temperature, the Earth's surface radiates energy with long wavelength. Collimated and diffuse components of radiation and their reflections, heat conduction and convection also occur on the Earth's surface. Without loss of generality, the major assumptions made are the following:

1. Carbon dioxide and water vapor are responsible for energy absorption in the troposphere in this model. Absorption bands of carbon dioxide are centered at 15, 4.3, 2.7, and 2 μm (see Fig. 1). The centers of absorption bands of water vapor can be considered at 71, 6.3, 2.7, 1.87 and 1.38 μm [23,24]. Major amounts of oxygen gas and nitrogen gas are transparent to infrared radiation.
2. Solar flux is governed by the Beer's law [39, 40]. Mie and Rayleigh scatterings of solar irradiation can be included in the extinction coefficient [23].
3. The diffuse component of radiation is solved by the widely used P_1_ approximation [23].
4. Heat transfer is unsteady and one-dimensional in the troposphere. This is because the troposphere layer is thinner than the affected region of the sun irradiation.
5. Fluid flow is neglected to a first approximation. Its effects on temperature near the Earth's surface can be accounted for by introducing the heat transfer coefficient often used in heat transfer field.

### 2.1 Absorption coefficients

Absorption coefficient can be effectively determined by an exponential wide band model [23, 24, 34].

(1) $$\kappa =\frac{\alpha }{\Delta \eta }\approx \frac{\tilde{\alpha }\rho_{CO_{2}}}{\tilde{\omega }A^{*}}\text{,}$$

where κ, α and Δη are, respectively, absorption coefficient, band intensity, and effective band width, whereas $\tilde{\alpha }$ and $\tilde{\omega }$ are correlation parameters of the wide band model. Eq. (1) indicates that an increase in band intensity or carbon dioxide density and a decrease in effective band width increases absorption coefficient. In this work, subscript of density $\rho_{CO_{2}}$ is eliminated for brevity. Dimensionless total band absorptance $A^{*}$ can be divided into Regions 1 through 3, governed by the following relationships:

For β≤1

(2) $$A^{*}=\tau_{0}\;\text{for}\;0\leq \tau_{0}\leq \beta$$

(3) $$A^{*}=2\sqrt{\tau_{0}\beta }-\beta ,\;\text{for}\;\beta \leq \tau_{0}\leq \frac{1}{\beta }$$

(4) $$A^{*}=\ln\left(\tau_{0}\beta \right)+2-\beta ,\;\text{for}\;\frac{1}{\beta }\leq \tau_{0}<\infty$$

where dimensionless optical thickness at the band center and overlap parameter are, respectively,

(5) $$\tau_{0}=\frac{\tilde{\alpha }\rho s}{\tilde{\omega }}\text{,}$$

(6) $$\beta \equiv \gamma P_{e}$$

Variables s and γ in Eqs. (5) and (6) are, respectively, optical path length and correlation parameter. Eqs. (2), (3), and (4) indicate that for a given overlap parameter dimensionless total band absorptance decreases increasing rate as optical thickness at the band center increases. Band intensity and correlation parameters in Eqs. (1) and (6) are given by

(7) $$\alpha ={\tilde{\alpha }}_{\text{0}}\text{(}\frac{\tilde{\alpha }}{{\tilde{\alpha }}_{\text{0}}}\text{)}\rho$$

(8) $$\tilde{\omega }={\tilde{\omega }}_{0}\sqrt{\frac{T}{T_{0}}}\text{,}\;{\text{T}}_{\text{0}}=100\;\text{K}$$

(9) $$\gamma =\gamma_{\text{0}}\text{(}\frac{\gamma }{\gamma_{\text{0}}}\text{)}$$

where T is temperature, correlation parameters ${\tilde{\alpha }}_{\text{0}}$, ${\tilde{\omega }}_{0}$ and $\gamma_{\text{0}}$ are listed in textbooks [23, 24]. The effective pressure accounting for collisions in Eq. (6) is

(10) $$P_{e}\equiv {\left[\frac{P_{a}}{p_{0}}\left(1+\left(b-1\right)x\right)\right]}^{n},\;p_{0}=1\;\text{atm}$$

where b and n are pressure parameters [23, 24], mole fraction of carbon dioxide is determined by Dalton's law

(11) $$x=\frac{p}{P_{a}}$$

The total pressure in Eq. (10) is specified as

(12) $$P_{a}=P_{E}e^{-0.00014\left(H-z\right)}$$

where atmospheric pressure at the Earth surface $P_{E}=1.01\times 10^{5}$ Pa.

### 2.2 Heat equations in atmosphere

With the above assumptions, the unsteady energy equation in the troposphere reduces to

(13) $$\frac{\partial \rho_{a}c_{p}T}{\partial t}=\frac{\partial }{\partial z}\left(k_{a}\frac{\partial T}{\partial z}\right)-\frac{\partial q_{c}}{\partial z}-\frac{\partial q_{d}}{\partial z}$$

where t is time, $\rho_{a}$, $c_{p}$ and $k_{a}$ are, respectively, density, specific heat and thermal conductivity of air. The term on the left-hand side of Eq. (13) represents unsteady change of internal energy, whereas terms on the right-hand side stand for heat conduction, radiative energy absorbed due to the collimated and diffuse components of radiation, respectively [23]. The collimated component in the second term on the right-hand side of Eq. (13) is governed by the Beer's law

(14) $$\frac{\partial q_{c}}{\partial z}=-\beta_{c}q_{c}$$

which absorption and scattering effects can be included in extinction coefficient $\beta_{c}$. The diffuse component in the last term of Eq. (13) is governed by [23].

(15) $$\frac{\partial {\text{q}}_{\text{d,ij}}}{\partial z}=\kappa_{ij}\left(4E_{b,j}-G_{d,ij}\right)$$

(16) $$\begin{array}{l} \frac{\partial G_{d,ij}}{\partial z}=-3\kappa_{ij}{\text{q}}_{d\text{,ij}}\\ \;\;\;\; \end{array}$$

where subscript ij represents species i and absorption band j, $E_{b}$ and $G_{d}$ are blackbody emissive power and incident radiation, respectively. Diffuse component yields

(17) $$q_{d}=\;\;\sum_{gas\;i,\;band\;j}q_{d,ij}=\sum_{band\;j}q_{d,CO_{2}\;j}+\sum_{band\;\text{j}}q_{d,H_{2}O\;j}$$

Boundary conditions of the diffuse radiation at the tropopause and Earth's surface are, respectively

(18) $$2q_{d,ij}=\frac{\varepsilon_{T}\left(4E_{b,j}-G_{d,ij}\right)}{2-\varepsilon_{T}}\;\text{at}\;z=0$$

(19) $$-2q_{d,ij}=\frac{\varepsilon \left(4E_{b,j}-G_{d,ij}\right)}{2-\varepsilon }\;\text{at}\;z=H$$

where $\varepsilon_{T}$ and ε are emissivities at the tropopause and Earth's surface, respectively. Energy balance at the Earth's surface is given by

(20) $$\;-k_{a}\frac{\partial T}{\partial z}=h_{E}\left(T_{E}-T_{g}\right)+h_{a}\left[T_{E}-T\left(9900,0\right)\right]-\left(1-\rho_{E}\right)\left(q_{c}+q_{d}\right)+\varepsilon_{E}\sigma T_{E}^{4}$$

where $\;h_{E}$ and $h_{a}$ are, respectively, heat transfer dissipates into the Earth ground and ambient air, $\;T_{E}$ and $T_{g}$ are temperature of the Earth's surface and that in the ground far from the Earth's surface, $\rho_{E}$, $\varepsilon_{E}$ are diffuse reflectivity and emissivity of the Earth's surface, and σ is Stefan-Boltzmann constant. The reference temperature T(9900) is chosen to be temperature nearly independent of heat transfer from the Earth's surface. The term on the left-hand side of Eq. (20) is heat conduction from the Earth's surface to air. Terms on the right-hand sides are heat transfer into the ground, convection to the troposphere, and absorption and emission of radiation by the Earth's surface, respectively. Boundary condition at the tropopause is

(21) $$\;k_{a}\frac{\partial T}{\partial z}=h_{a\infty }\left(T-T_{\infty }\right)$$

where $\;h_{a\infty }$ and $T_{\infty }$ are, respectively, heat transfer coefficient and temperature at the tropopause.

## 3 Results and discussion

In this work, absorption coefficients in different absorption bands of carbon dioxide responsible for determination of temperature profiles across the troposphere are predicted. The absorption coefficient is calculated by using the exponential wide band model governed by Eqs. (1), (2), (3), (4), (5), (6), (7), (8), (9), (10), (11), and (12) and updated at each time. Temperature is determined from Eqs. (13), (14), (15), (16), and (17) together with boundary conditions (18), (19), (20), and (21).

### 3.1 Comparison with available experimental and theoretical data

**Fig. 3:**
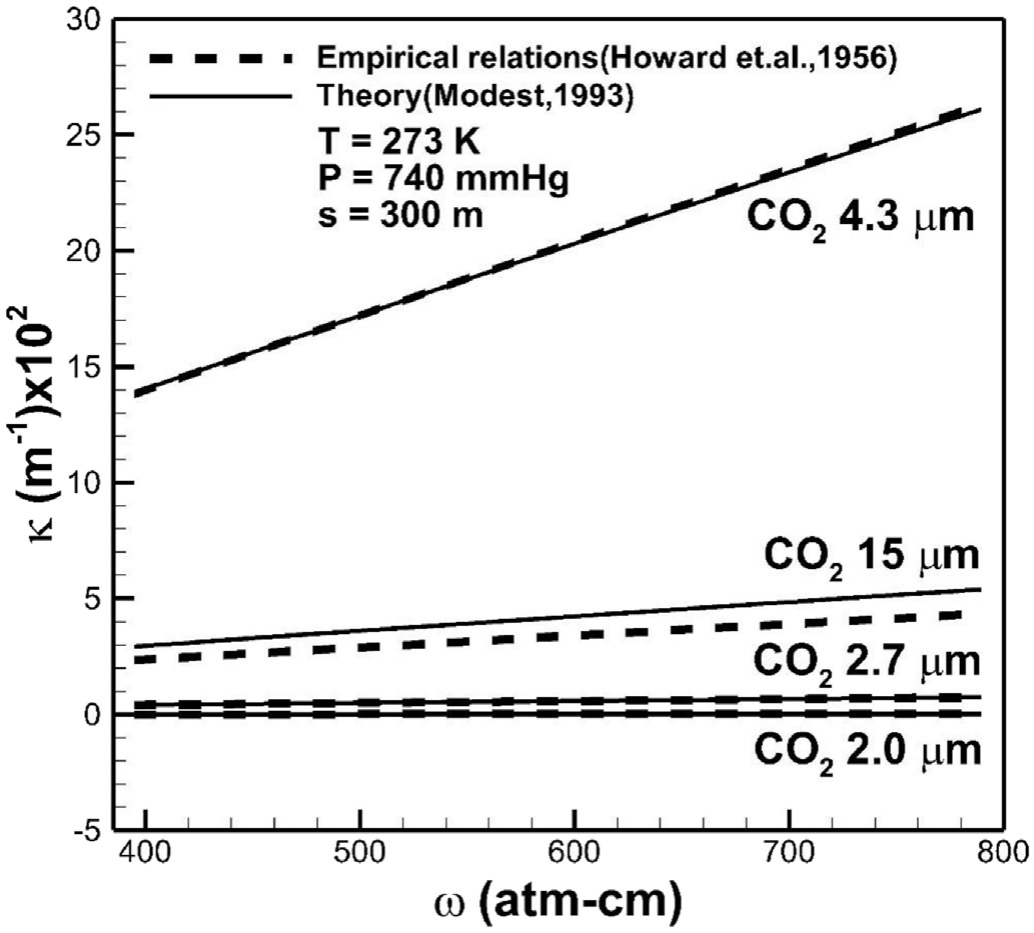
Comparison of absorption coefficient of carbon dioxide in bands with wavelength centered at 15, 4.3, 2.7, and 2 μm, predicted from available theory [29] and exponential wide band model from this work [23]. Abscissa for 1 atm-cm = $2.69\times 10^{19}\;{\text{molecules/cm}}^{2}$ at STP. An increase in concentration of carbon dioxide increases absorption coefficient.

**Fig. 4:**
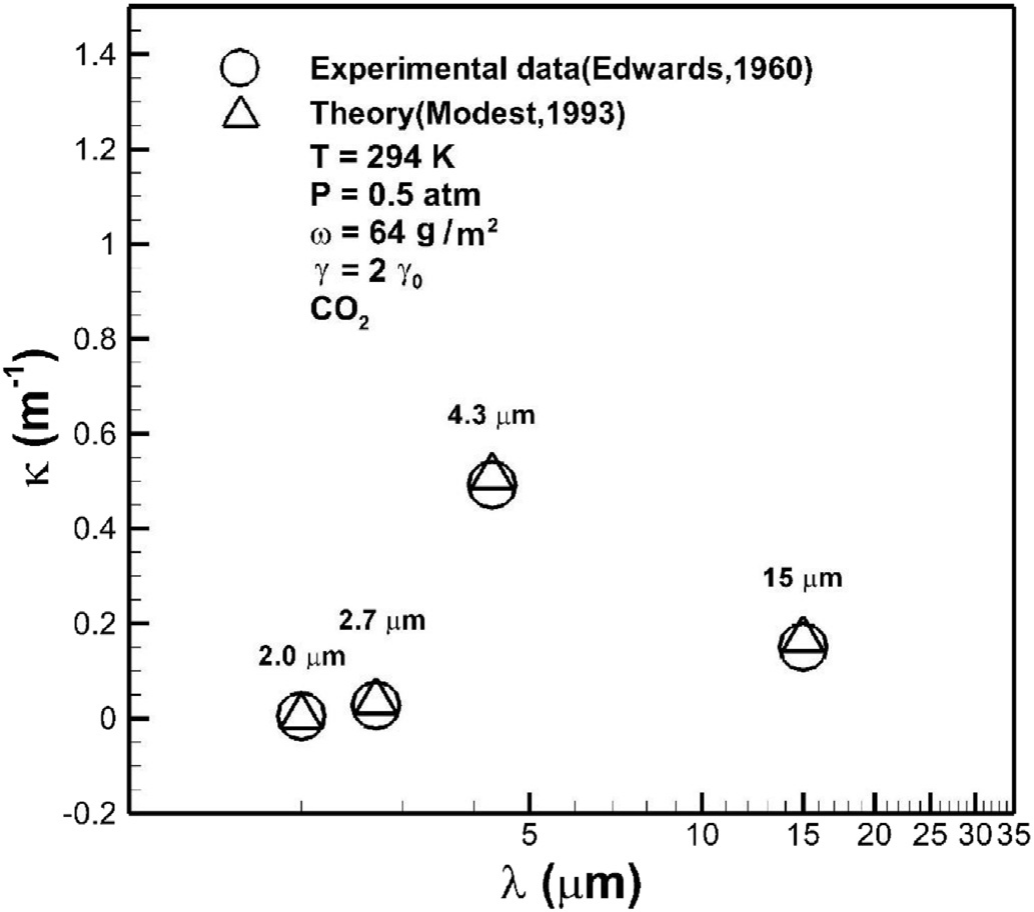
Comparison between predicted results based on the exponential wide band model [23] and measurement data [41] of averaged absorption coefficients in different bands of carbon dioxide.

In the case of temperature of 273 K, total pressure of 740 mmHg, and optical path length of 300 m, Fig. 3 shows good comparison between the predicted absorption coefficient of carbon dioxide in bands with wavelength centered at 15, 4.3, 2.7, and 2 μm from this work using exponential wide band correlation [23] and available correlation from experimental data [29]. Deviation between two theoretical models can be attributed to the chosen optical path length, as can be seen later. The abscissa in unit of atm-cm is a measure of the total number of carbon dioxide absorbers per unit area traversed by the beam of radiation. Based on the Avogadro's hypothesis, molecular weight M in grams in any gas occupies 22.4 liters and $6.02\times 10^{23}$ gas molecules at STP. Since 1 atm-cm is proportional to number of particles in a length of 1 cm of gas per unit cm^2^, 1 atm-cm = $6.02\times 10^{23}/2.24\times 10^{4}$ = $2.69\times 10^{19}\;{\text{molecules/cm}}^{2}$ at STP. An increase in concentration of carbon dioxide increases absorption coefficient. Fig. 4 shows measured and predicted average absorptions in different bands of carbon dioxide [41]. Agreement between predicted and measured results is quite good. The highest and second highest absorption coefficients are, respectively, in bands centered at 4.3 μm and 15 μm.

### 3.2 Absorption coefficients affected by different optical path lengths

**Fig. 5:**
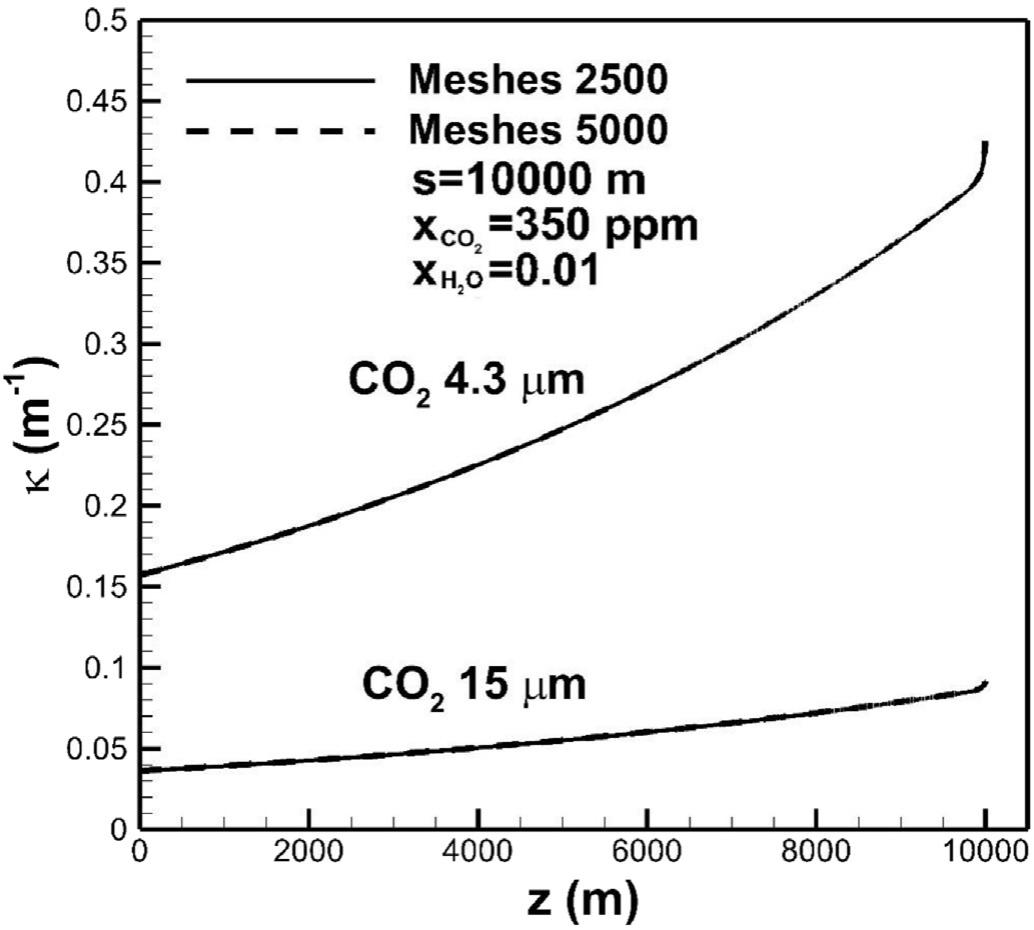
Convergence test of absorption coefficients of carbon dioxide in bands centered at 15 and 4.3 μm across the troposphere for different grid sizes.

Fig. 5 show convergence test of two highest absorption coefficients in bands centered at 15 and 4.3 μm of carbon dioxide across the troposphere layer for different grid sizes. Number of meshes greater than 2500 can give good results. Meshes were chosen to be dense near the Earth's surface.

**Fig. 6:**
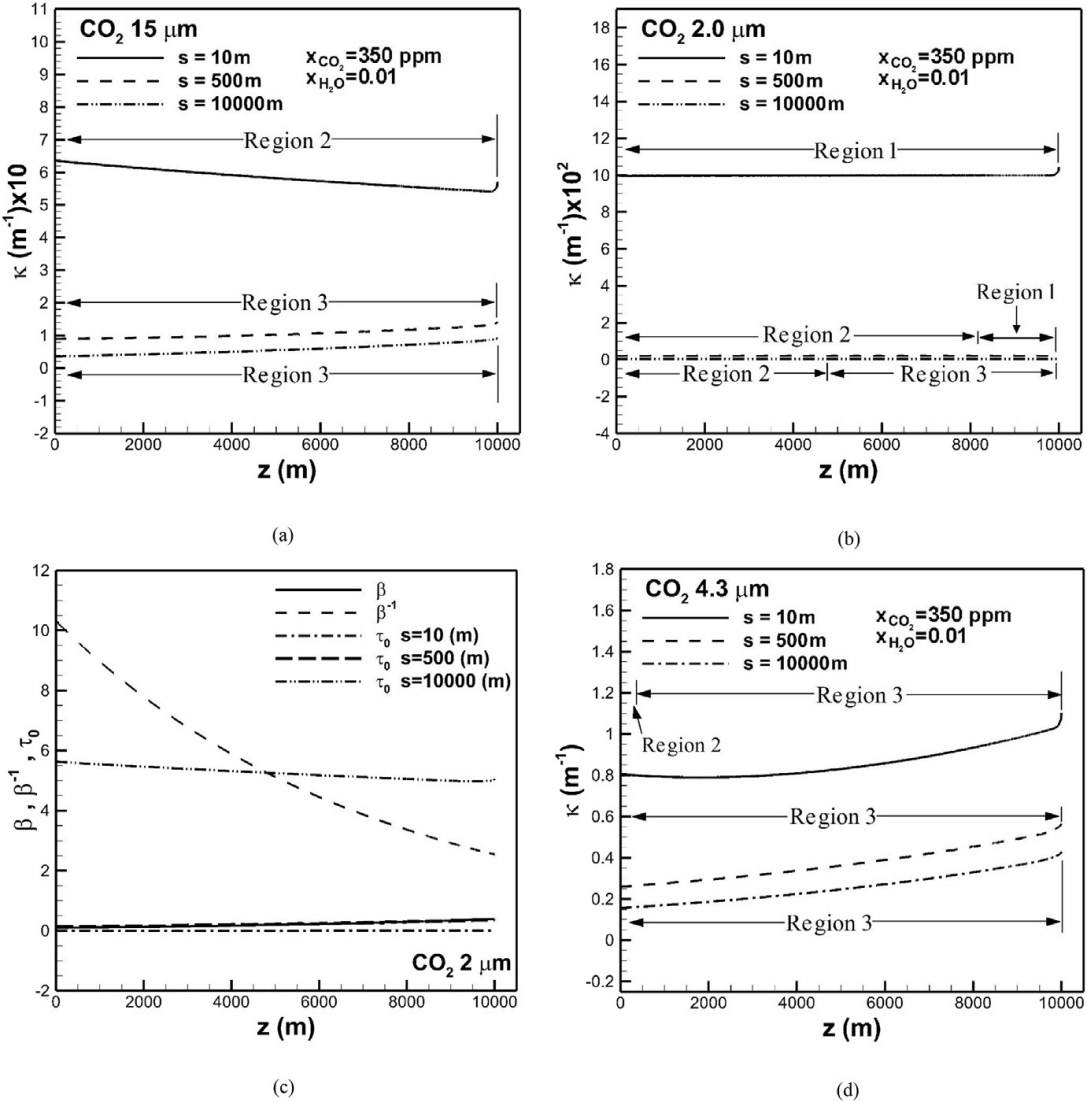
The effects of optical path length on absorption coefficients of bands centered at (a) 15 μm, (b) 2 μm, and optical thickness at band center, overlap parameter and its reciprocal at band centered at (c) 2 μm, and absorption coefficient of band centered at (d) 4.3 μm across the troposphere. Regions 1–3 are defined by overlap parameter in Eqs. (2), (3), and (4).

Specification of optical path length is required to determine dimensionless optical thickness at the band center (see Eq. (5)) and absorption coefficient. Dimensionless optical thickness at the band center for different optical path length can be categorized by three regions and governed by Eqs. (2), (3), and (4), where the overlap parameter β is smaller than unity. Small overlap parameter is attributed to small values of correlation parameter $\gamma_{\text{0}}$ and partial pressure of carbon dioxide (see Eqs. (6), (9), and (10)). Fig. 6(a) shows the effects of optical path length on absorption coefficient in the band centered at wavelength of 15 μm subject to carbon dioxide concentration of 350 ppm and water vapor concentration of 0.01. For a small optical path length of 10 m Region 2 governed by Eq. (3) is prevailed in the entire troposphere. Absorption coefficient is as high as 0.5 m^−1^. As optical path length increases to s = 500 m and $10^{4}$ m, only Region 3 governed by Eq. (4) is prevailed in the troposphere layer. Absorption coefficient, however, decreases to be less than 0.1 m^−1^. Since an increased optical path length increases optical thickness at the band center, dimensionless total band absorptance increases. As a result, effective band width increases whereas absorption coefficient decreases.

In the absorption band centered at 2.0 μm Fig. 6(b) shows that absorption coefficient across the troposphere subject to a small optical path length of 10 m in Region 1 is a relative constant of 0.1 m^−1^. It can be confirmed by referring to Eqs. (1), (2), and (5), leading to absorption coefficient κ=1/s = 0.1 m^−1^. Owing to independence of radiative properties, absorption coefficient in Region 1 is irrelevant [22, 23, 24]. Similarly, an increase in optical path length decreases absorption coefficient. Regions 1 and 2 for an optical path length of 500 m occur in the lower and upper regions of the troposphere, respectively. Absorption coefficient is irrelevant in the lower region of troposphere. An optical path length of $10^{4}$ m results in Regions 2 and 3 in the upper and lower troposphere, respectively. It is worthy of mentioning that even though carbon dioxide density increases in the direction toward the Earth's surface, Region 1 near the Earth's surface for optical path length of 500 m is attributed to an increase in overlap parameter, as shown in Fig. 6(c). The overlap parameter which is independent of optical path length increases due to an increase in partial pressure of carbon dioxide in the direction toward the Earth's surface (see Eqs. (6) and (10)). It can be seen that optical thickness at the band center is comparatively constant. Region 1 is below the overlap parameter across the troposphere for optical path length of 10 m. Regions 2 and 1 in the upper and lower troposphere subject to optical path length of 500 m is because optical thickness at the band center is from above to below the overlap parameter. For optical path length of $10^{4}$ m Regions 2 and 3 in the upper and lower troposphere are, respectively, attributed to that optical thickness at the band center is between the overlap parameter and reciprocal of overlap parameter, and above reciprocal of the overlap parameter.

In the band centered at 4.3 μm Fig. 6(d) shows that Regions 2 and 3 occur in the upper and lower regions of the troposphere, respectively, for an optical path length of 10 m. Region 2 with small optical thickness at the band center is resulting from low density of carbon dioxide. Similarly, Region 3 occupies the entire troposphere as optical path length increases to 500 m and $10^{4}$ m. Referring to Fig. 6(a) and (d) show that Region 3 occurs in the entire troposphere in the major absorption bands centered at 15 μm and 4.3 μm with high optical path lengths of 500 m and $10^{4}$ m. High values of optical path length in Region 2 or 3 represent less absorption or small absorption coefficient due to molecules behaved as a rigid rotator and nonrigid rotator [32, 38]. Relevant values for the path length were as high as around 5.5 km as suggested by Ceballos et al. [40], McClatchey et al. [42], Smirnov [43], and Shaw [44].

**Fig. 7:**
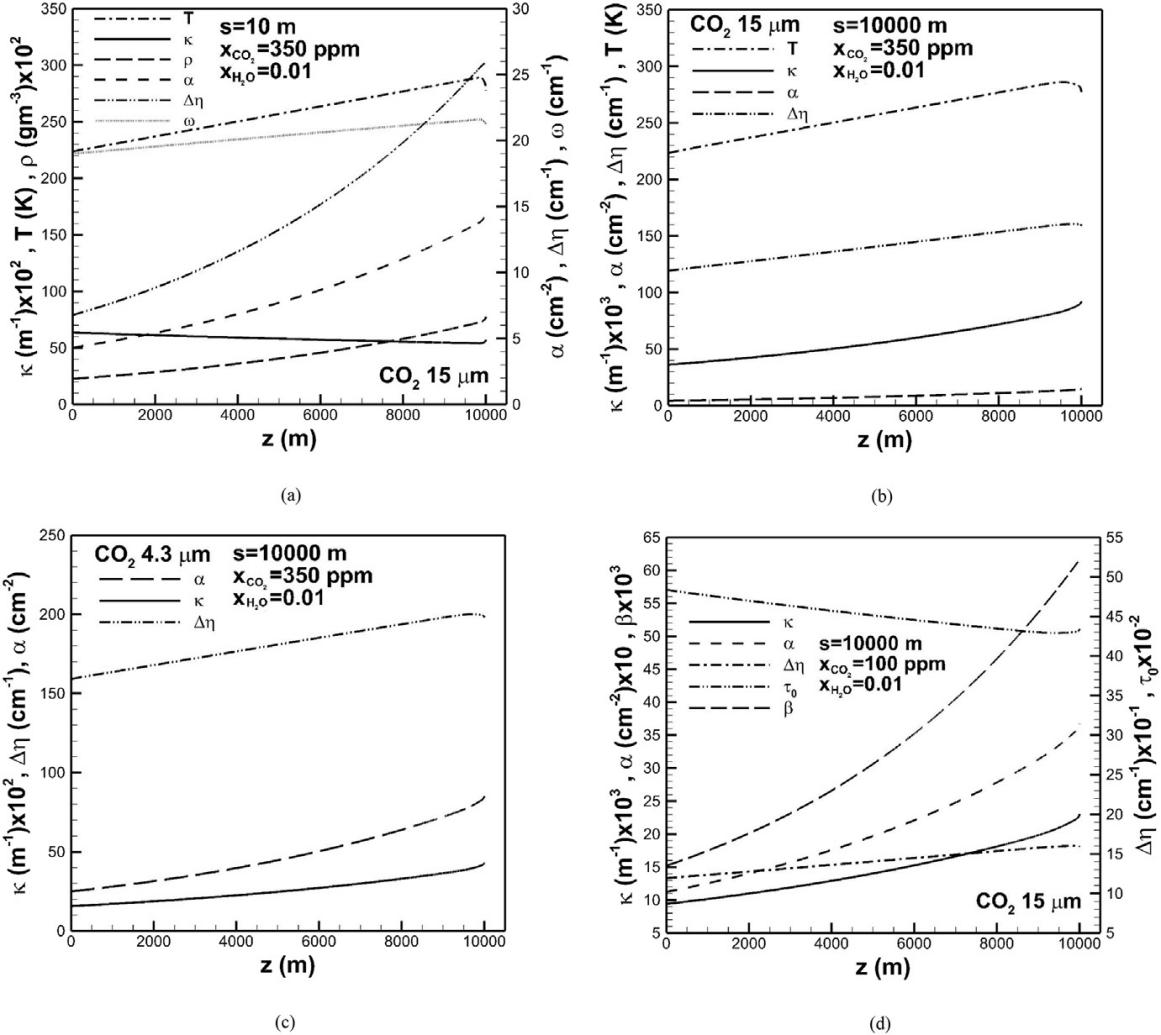
Absorption coefficient, temperature, density, band intensity, correlation parameter related to band width and effective width of bands centered at (a) 15 μm for s =10 m, absorption coefficient, temperature, band intensity and effective width of bands centered at (b) 15 μm for s = $10^{4}$ m, absorption coefficient, band intensity and effective width of bands centered at (c) 4.3 μm for s = $10^{4}$ m and carbon dioxide concentration of 350 ppm, and absorption coefficient, band intensity, optical thickness at band center, overlap parameter and effective width of bands centered at (d) 15 μm for s = $10^{4}$ m and carbon dioxide concentration of 100 ppm across the troposphere.

For a clear interpretation absorption coefficient, temperature, carbon dioxide density, band intensity, effective band width, and correlation parameter related to band width across the troposphere subject to an optical path length of 10 m in Region 2 are shown in Fig. 7(a). The band is centered at 15 μm with carbon dioxide concentration of 350 ppm and water vapor concentration of 0.01. It shows that carbon dioxide density, band intensity and effective band width monotonically increase in the direction toward the Earth's surface. Temperature and correlation parameter related to band width, however, exhibit increase and then decrease in the direction toward the Earth's surface. Differentiating effective band width given by $\Delta \eta =\tilde{\omega }A^{*}$ from Eq. (1) with respect to depthwise position and substituting Eqs. (2), (3), and (4) lead to

(22) $$\frac{d\Delta \eta }{dz}=\frac{\tilde{\omega }\tau_{0}}{\rho }\frac{d\rho }{dz}\;\text{for}\;0\leq \tau_{0}\leq \beta$$

(23) $$\frac{d\Delta \eta }{dz}=\frac{\tilde{\omega }}{\rho }\sqrt{\beta \tau_{0}}\frac{d\rho }{dz}+\left(\sqrt{\beta \tau_{0}}-\beta \right)\frac{\tilde{\omega }}{2T}\frac{dT}{dz}\;\text{for}\;\beta \leq \tau_{0}\leq \frac{1}{\beta }$$

(24) $$\frac{d\Delta \eta }{dz}=\frac{\tilde{\omega }}{\rho }\frac{d\rho }{dz}+\left(\ln\sqrt{\beta \tau_{0}}+1-\beta \right)\frac{\tilde{\omega }}{2T}\frac{dT}{dz}\;\text{for}\;\frac{1}{\beta }\leq \tau_{0}<\infty$$

Eqs. (22), (23), and (24) show that an increase in optical thickness at the band center, respectively, enhances and reduces the effects of temperature and carbon dioxide density on effective band width. Therefore, effective band width in Region 2 with a small optical thickness at the band center exponentially increases in the direction toward the Earth's surface for a significant increase in carbon dioxide density, even though temperature drops near the Earth's surface. A decreased absorption coefficient is due to an exponential increase in effective band width toward the Earth's surface.

Absorption coefficient, temperature, band intensity, effective width of band centered at 15 μm and optical path length of $10^{4}$ m across the troposphere are shown in Fig. 7(b). In contrast to previous Fig. 7(a), an increase in optical thickness at the band center results in effective band width to increase linearly and then decrease in the direction toward the Earth's surface. This is attributed to the dominant effect of temperature on effective band width subject to a higher optical thickness at the band center. A rapid increase in absorption coefficient in the direction toward and near the Earth’ surface is attributed to the rapid increase in band intensity as well as a linear increase and then decrease in effective band width. Similar trends of absorption coefficient, band intensity, and effective band width across the troposphere can be found in the band centered at 4.3 μm and optical path length of $10^{4}$ m, as shown in Fig. 7(c). Referring to Fig. 7(b), Fig. 7(d) shows that a decrease in carbon dioxide concentration decreases absorption coefficient. This is attributed to a decrease in band intensity whereas effective band width maintains nearly the same order of magnitude. Since optical thickness at the band center is greater than reciprocal of the overlap parameter, Region 3 is prevailed in the troposphere. Temperature also plays an important role in effective band width. Spatial variation of absorption coefficient thus decreases.

### 3.3 Absorption coefficients for different carbon dioxide concentrations

**Fig. 8:**
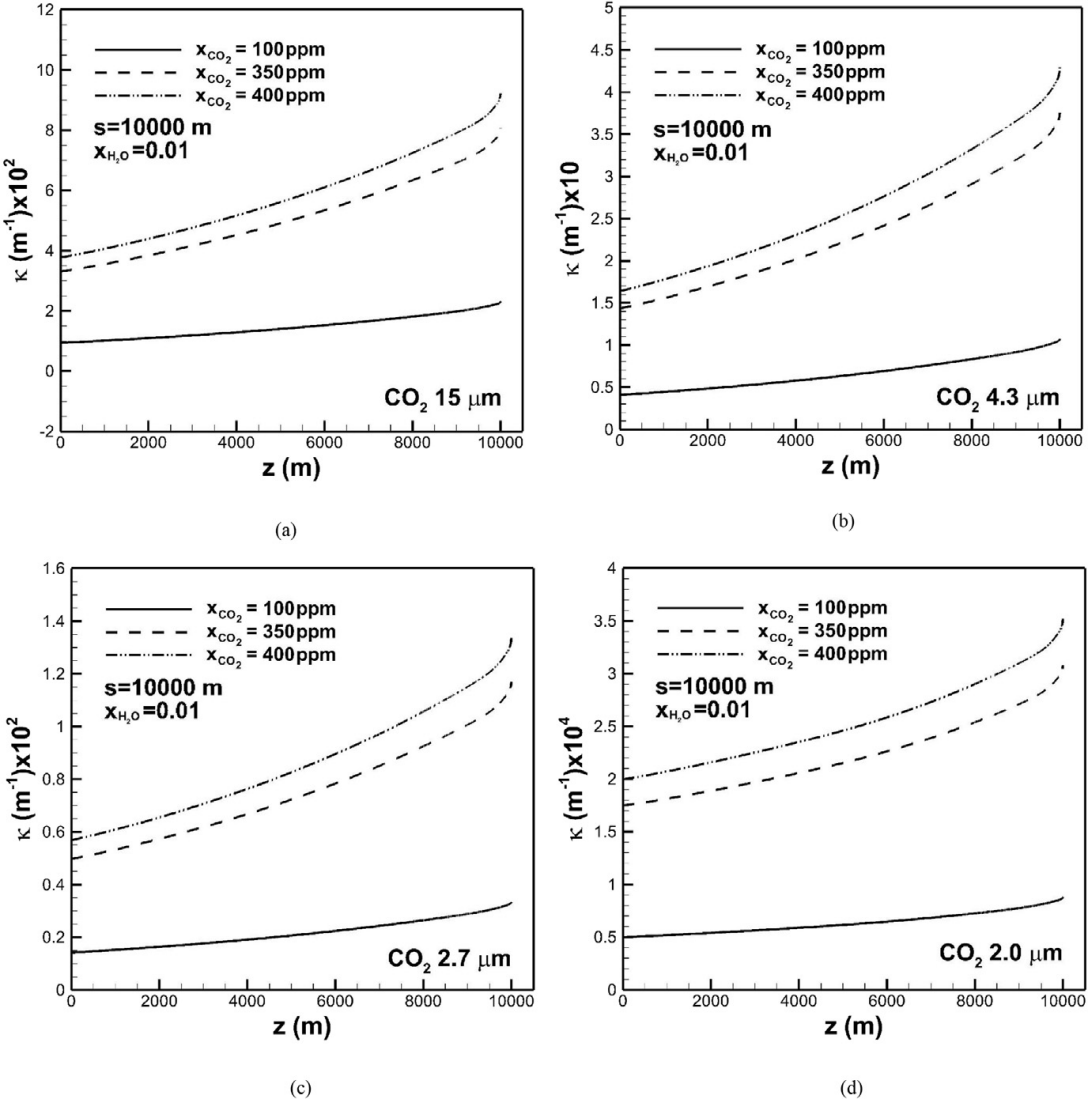
The effects of carbon dioxide concentration on absorption coefficient across the troposphere in different bands centered at (a) 15 μm, (b) 4.3 μm, (c) 2.7 μm, and (d) 2 μm. An increase in carbon dioxide concentration significantly enhances change in absorption coefficient across troposphere.

The effects of carbon dioxide concentration on absorption coefficient subject to optical path length of $10^{4}$ m in the band centered at 15 μm and water vapor of 0.01 are shown in Fig. 8(a). Absorption coefficient increases significantly as carbon dioxide concentration increases. This is attributed to an exponential increase in band intensity or density of carbon dioxide, and a linear increase in effective band width affected by temperature, as mentioned previously. Trends of absorption coefficients through the troposphere for different concentrations are similar. In the case of carbon dioxide concentration of 100 ppm, absorption coefficient increases from 0.01 m^−1^ at the tropopause to 0.025 m^−1^ at the Earth's surface. For a carbon dioxide concentration of 400 ppm absorption coefficient increases from 0.038 m^−1^ at the tropopause to 0.095 m^−1^ at the Earth's surface. The variation of absorption coefficient across the troposphere is significant for a high concentration of carbon dioxide. Fig. 8(b) shows that absorption coefficients in the band centered at 4.3 μm increase from 0.04 m^−1^ and 0.165 m^−1^ at the tropopause to 0.11 m^−1^ and 0.44 m^−1^ at the Earth's surface for carbon dioxide concentrations of 100 and 400 ppm, respectively. Absorption coefficients in the band centered at 4.3 μm are greater than those in band centered at 15 μm. Absorption coefficients of band centered at 2.7 and 2 μm exhibit similar trends, as shown in Fig. 8(c) and (d). However, absorption coefficients decrease.

### 3.4 Temperature profile accounting for absorption coefficient across troposphere

**Fig. 9:**
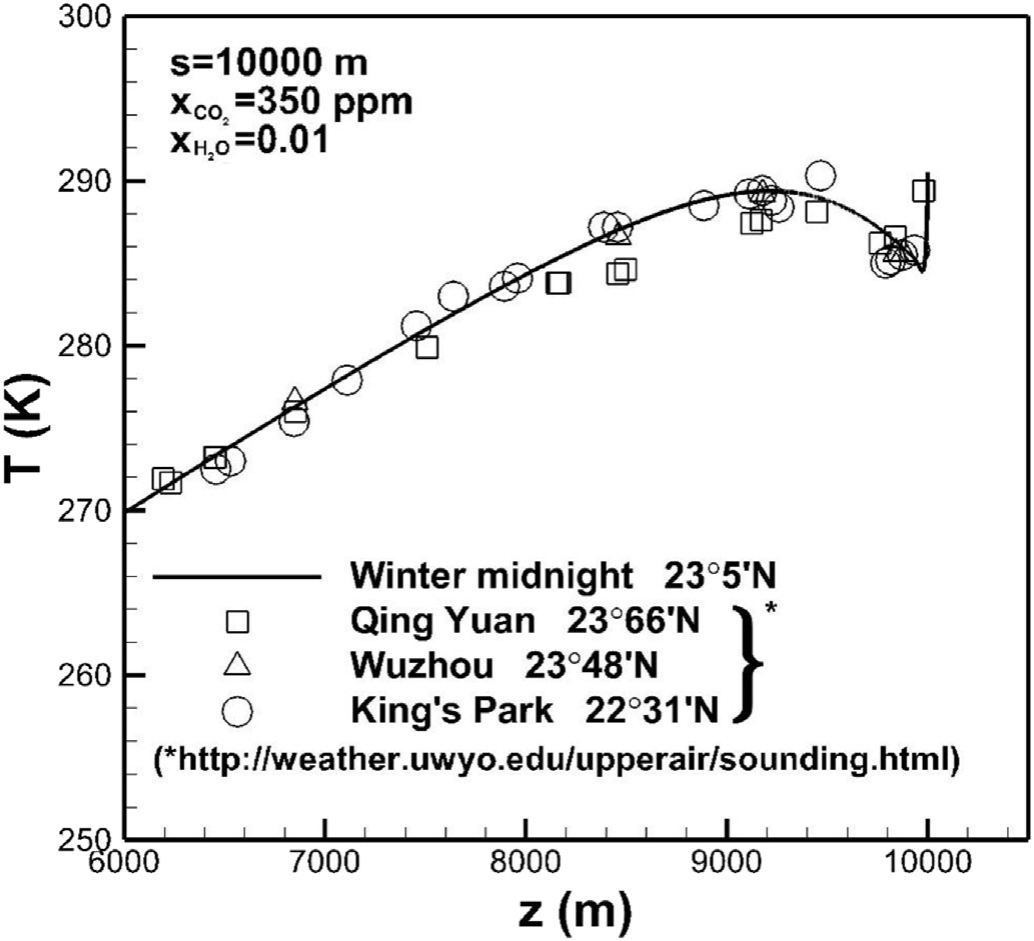
Comparison of a temperature profile across the troposphere between experimental data and this work.

A comparison of temperature profile across the troposphere at a noon of winter between experimental data and this work is shown in Fig. 9. Three locations are approximately at identical latitude. There exist a local minimum and maximum near locations around 9,990 m and 9,200 m, respectively. Temperature exhibits the maximum value at the Earth's surface, since the high absorption coefficient resulting in strong absorption of solar irradiation at noon.

## 4 Conclusions

In this work, the predicted temperature increases in the direction toward the Earth's surface until locations near the Earth's surface. Carbon dioxide density exponentially increases in the direction toward the Earth's surface. The conclusions drawn are the following:

1. Absorption coefficient across the troposphere layer for different carbon dioxide concentrations and optical path lengths are systematically presented. A systematical and detailed study of absorption coefficient based on the exponential wide band model is essentially required for a reliable prediction of temperature profiles across the troposphere.
2. Absorption coefficient determined by the ratio between band intensity and effective band width in each band increases as the Earth's surface is approached. This is because the increase in band intensity is greater than that in effective band width. A rapid increase in absorption coefficient near the Earth's surface is attributed to that the effective band width can become decreased.
3. Band intensity increase with carbon dioxide density. The effect of temperature on band intensity is negligibly small. An increase in optical path length, respectively, enhances and reduces the effects of temperature and carbon dioxide density on effective band width. Effective band width thus exhibits exponential and linear increases for small and high optical path lengths, respectively.
4. Absorption coefficient is increasingly influenced from carbon dioxide density to temperature in the direction toward the Earth's surface.
5. Absorption coefficient decreases as optical path length increases. Relevant values of optical path length should be beyond 5000 m in different bands. Unknown optical path length requires experimental measurements.
6. The first and second highest absorption coefficients are confirmed to occur, respectively, in absorption bands centered at 4.3 μm and 15 μm.
7. An increase in carbon dioxide concentration increases the difference in absorption coefficient between the tropopause and Earth's surface. Absorption coefficients in the band centered at 4.3 μm increase from 0.04 m^−1^ and 0.165 m^−1^ at the tropopause to 0.11 m^−1^ and 0.44 m^−1^ at the Earth's surface for carbon dioxide concentrations of 100 and 400 ppm, respectively.
8. Absorption coefficient in Region 1 reduces to a reciprocal of optical path length, which is irrelevantly independent of radiative properties.

## Declarations

### Author contribution statement

Peng-Sheng Wei: Conceived and designed the experiments; Analyzed and interpreted the data; Wrote the paper.

Yin-Chih Hsieh, Da-Lun Yen: Conceived and designed the experiments; Performed the experiments; Analyzed and interpreted the data.

Hsuan-Han Chiu, Chieh Lee, Yi-Cheng Tsai, Te-Chuan Ting: Conceived and designed the experiments; Performed the experiments; Analyzed and interpreted the data; Contributed reagents, materials, analysis tools or data.

### Funding statement

This research did not receive any specific grant from funding agencies in the public, commercial, or not-for-profit sectors.

### Competing interest statement

The authors declare no conflict of interest.

### Additional information

No additional information is available for this paper.
